# Deafness-in-a-dish: modeling hereditary deafness with inner ear organoids

**DOI:** 10.1007/s00439-021-02325-9

**Published:** 2021-08-03

**Authors:** Daniel R. Romano, Eri Hashino, Rick F. Nelson

**Affiliations:** Department of Otolaryngology-Head and Neck Surgery, Indiana University School of Medicine, 980 W. Walnut Street, WH-C426C, Indianapolis, IN 46202 USA

## Abstract

Sensorineural hearing loss (SNHL) is a major cause of functional disability in both the developed and developing world. While hearing aids and cochlear implants provide significant benefit to many with SNHL, neither targets the cellular and molecular dysfunction that ultimately underlies SNHL. The successful development of more targeted approaches, such as growth factor, stem cell, and gene therapies, will require a yet deeper understanding of the underlying molecular mechanisms of human hearing and deafness. Unfortunately, the human inner ear cannot be biopsied without causing significant, irreversible damage to the hearing or balance organ. Thus, much of our current understanding of the cellular and molecular biology of human deafness, and of the human auditory system more broadly, has been inferred from observational and experimental studies in animal models, each of which has its own advantages and limitations. In 2013, researchers described a protocol for the generation of inner ear organoids from pluripotent stem cells (PSCs), which could serve as scalable, high-fidelity alternatives to animal models. Here, we discuss the advantages and limitations of conventional models of the human auditory system, describe the generation and characteristics of PSC-derived inner ear organoids, and discuss several strategies and recent attempts to model hereditary deafness in vitro. Finally, we suggest and discuss several focus areas for the further, intensive characterization of inner ear organoids and discuss the translational applications of these novel models of the human inner ear.

## Introduction

An estimated 430 million people worldwide (13 million in the United States) have moderate-to-profound hearing loss (GBD Hearing Loss Collaborators 2021; Goman and Lin 2016). Hearing loss is not only a quality-of-life issue, with hearing impaired persons reporting feelings of isolation, frustration, and anxiety (Khan et al. 2020; Lindburg et al. 2021), but also a significant contributor to the global disability burden (GBD Hearing Loss Collaborators 2021). Reduced hearing is associated with language and other developmental delay (Figueras et al. 2008; Tomblin et al. 2015), cognitive decline (Lin et al. 2013), and depression (Li et al. 2014), with working-age adults demonstrating lower average wages and reduced labor force participation (Jung and Bhattacharyya 2012) and elderly persons reporting increased difficulty in completing activities of daily living (Dalton et al. 2003; Gopinath et al. 2012). Most permanent hearing loss is of the sensorineural type (SNHL). Causes of SNHL include aging (Agrawal et al. 2008; Yamoah et al. 2020), infection (Bedford et al. 2001; Brown et al. 2009; Goderis et al. 2014), noise exposure (Lie et al. 2016), ototoxic drugs (Farzal et al. 2016; Frisina et al. 2016), traumatic disruption of the otic capsule (Honeybrook et al. 2017), and a long—and growing—list of single-gene mutations (Shearer et al. 1993; Toriello and Smith 2013). Regardless of the specific etiology, all SNHL ultimately results from the loss, dysfunction, or malformation of cochlear hair cells, spiral ganglion neurons, and/or the synapses in between. Though the causes of SNHL are generally well established, the underlying pathophysiologic mechanisms of even the most common causes remain poorly elucidated at the cellular and molecular level.

The successful development of new therapeutic approaches, such as growth factor, stem cell, and gene therapies, will require a yet deeper understanding of the biology of hearing and deafness, as well as high-fidelity models for pre-clinical testing. Since the human inner ear cannot be biopsied without causing significant, irreversible damage to the hearing or balance organ, the biological study of human inner ear cells has traditionally been limited to scarce fetal or cadaveric tissues. Thus, much of our current understanding of the cellular and molecular biology of human deafness, and of the human auditory system more broadly, has been inferred from observational and experimental studies in animal models [e.g., mouse (*Mus musculus*), chicken (*Gallus gallus*), zebrafish (*Danio rerio*), and African clawed frog (*Xenopus laevis*)]. Scientists have developed over 50 mouse models of hereditary deafness (Friedman et al. 2007), as well as rodent models for cochlear toxicity (Fernandez et al. 2019), noise-induced hearing loss (Escabi et al. 2019; Holt et al. 2019), infection (Yun et al. 2015), age-related hearing loss (presbycusis) (Cai et al. 2018; Hunter and Willott 1987), and cochlear ischemia, which is implicated in sudden SNHL (Gyo 2013). The anatomical and histological similarity of the human and rodent inner ears has also made rodents useful for modeling the many technical and biological hurdles to stem cell and gene therapy in the inner ear (Al-Moyed et al. 2019; Chen et al. 2012; Gyorgy et al. 2019; Pandit et al. 2011).

**Fig. 1:**
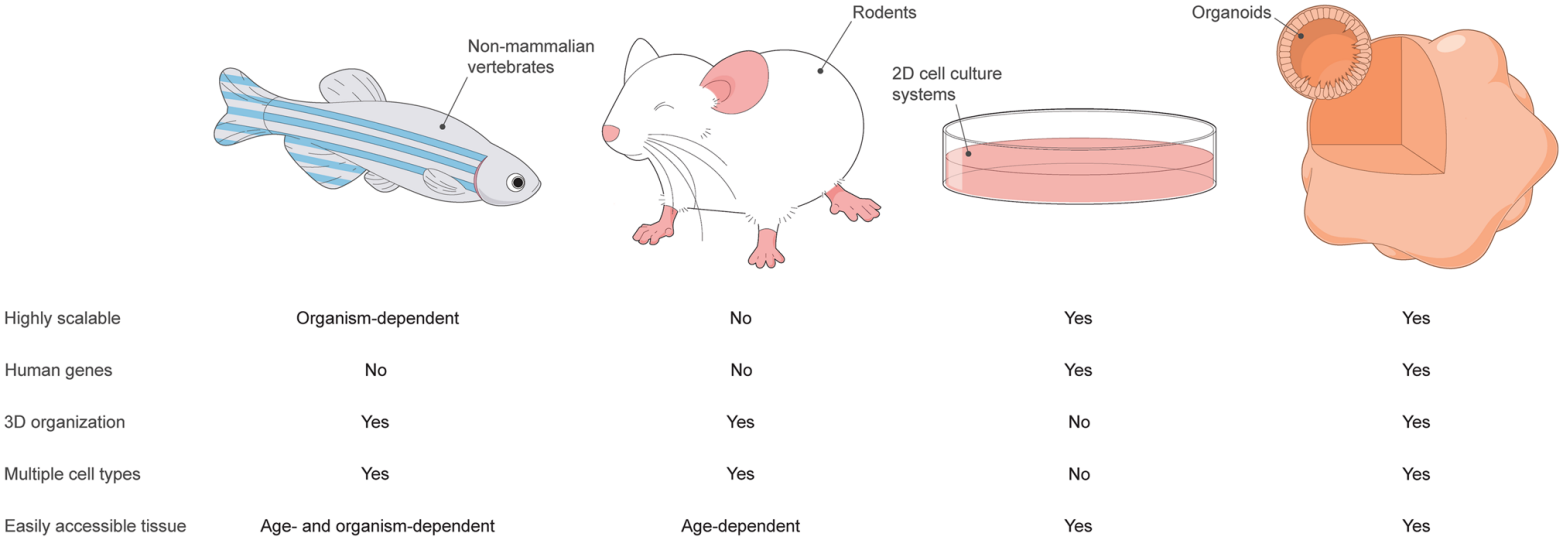
Chart comparing the characteristics of non-mammalian vertebrate (left), rodent (left-middle), 2D cell culture (right-middle), and organoid (right) models of the human auditory system

While each animal model has its own advantages and limitations (Fig. 1), all exhibit important differences from the human auditory system (Fig. 1). The hair cells of zebrafish and other non-mammalian vertebrates display a robust spontaneous regenerative response not seen in the mammalian organ of Corti (Corwin and Cotanche 1988; Harris et al. 2003; Roberson and Rubel 1994; Ryals and Rubel 1988; Schuck and Smith 2009), while those of the rodent cochlea do not acquire an adult-like morphology until the early postnatal period (Lenoir et al. 1987), in contrast to the appearance of adult-like hair cells in the third trimester of human gestation (Lavigne-Rebillard and Pujol 1986). Indeed, the stages of mouse and human inner ear development are hardly equivalent (Yamoah et al. 2020). In addition, while more than 99% of genes in the mouse genome have a human homologue (with approximately 80% having a one-to-one orthologue) (Mouse Genome Sequencing Consortium 2002), the targeted introduction of human deafness-related mutations into the mouse genome does—in some cases—fail to produce a deafness phenotype (Lu et al. 2014; Tona et al. 2020), suggesting that the sequence homology of a gene does not necessarily translate to functional identicality of its end-product. This was demonstrated more systematically by Liao and Zhang (2008), who found that more than 20% of a sample of one-to-one mouse orthologues of human essential genes (i.e., those genes required for survival to reproductive age or reproduction itself) were non-essential.

## In vitro generation of inner ear tissue

An alternative and complementary approach to generating animal models is the in vitro derivation of inner ear tissues from mouse or human pluripotent stem cells (PSCs) or tissue-specific progenitor cells. Specialized tissues are generated from PSCs through a process termed directed differentiation, which involves the precisely timed addition of growth factors and small molecules to recapitulate the signaling events of in vivo development. Culture systems are more scalable than animal models, and cells in vitro can be easily accessed for electrophysiological, molecular, and imaging studies (Fig. 1). PSCs and tissue-specific progenitor cells can be distinguished by their respective capacities for cell fate specification (virtually unlimited versus lineage-restricted), and in theory, self-renewal (infinite versus limited). However, PSCs may spontaneously differentiate in culture, and researchers have demonstrated using multiple cytogenetic methods that, over long-term culture, PSCs have a tendency to accumulate chromosomal aberrations (Dekel-Naftali et al. 2012; Martins-Taylor 2011; Merkle et al. 2017; Narva et al. 2010; International Stem Cell Initiative 2011; Yang et al. 2010), which, in turn, have been shown to affect their differentiation capacity (Fazeli et al. 2011; Markouli et al. 2019; Yang et al. 2010). The combination of spontaneous differentiation and accumulated chromosomal aberrations likely imposes a de facto passaging limit on PSCs in culture.

From a culturing standpoint, there are at least two important differences between mouse and human PSCs. First, in vitro tissues arise more slowly from human PSCs (hPSCs) than from mouse PSCs (mPSCs) (Compare Koehler et al. 2013, 2017), reflecting the longer gestational period in humans (280 days versus 20 days). Second, mPSCs and hPSCs have distinct states of pluripotency—the ground (naïve) and primed states, respectively (Nichols and Smith 2009). The ground state is embodied by the inner cell mass of the pre-implantation mouse blastocyst, while the primed state is embodied by the epiblast of the post-implantation mouse embryo. Mouse induced PSCs (miPSCs), like mESCs, exhibit the properties of naïve pluripotency. Conversely, hPSCs, even when derived as hESCs from the pre-implantation inner cell mass, more closely resemble post-implantation mouse epiblast cells (Brons et al. 2007; Tesar et al. 2007) and are, therefore, primed PSCs. This difference is important, as primed cells demonstrate poorer survival in single-cell suspension and are poised to differentiate along certain lineages. Together, these differences generally lead to the slower, less efficient, and more inconsistent in vitro derivation of inner ear tissues from hPSCs than from mESCs.

Despite the advantages of using mPSCs, in vitro models of the mouse inner ear obviously do not provide the ability to research human tissues. Conversely, hPSC-based culture systems allow researchers to study auditory system development, structure, physiology, and regeneration in living tissue that is genetically identical to the human inner ear. However, the transcriptional similarity of these models is highly dependent on the fidelity of the culture system. Conventional 2D culture systems, in which cells are grown on a glass or plastic substrate, have generally failed to yield transcriptionally, morphologically, and physiologically mature hair cell-like cells in appreciably high numbers (Chen et al. 2012; Ealy et al. 2016; Oshima et al. 2010; Ouji et al. 2012; Ronaghi et al. 2014). Indeed, it is likely that the 2D microenvironment is unable to accurately recapitulate the cell–cell and cell–ECM (extracellular matrix) interactions seen in vivo, which provide important cues for differentiation and gene expression. The use of utricular feeder cells or stromal conditioned medium can overcome this limitation to some degree (Oshima et al. 2010; Ouji et al. 2012), but these practices can lead to highly variable cultures. There is also a second, inherent limitation to modeling SNHL in 2D culture. That is, 2D culture systems are—by definition—incapable of modeling the complex, 3D process of mammalian inner ear morphogenesis or the precise, 3D spatial organization of the adult inner ear and its embryologic forerunners.

It is perhaps unsurprising, then, that 3D culture systems can overcome many of the limitations of 2D culture, resulting in higher fidelity models (Fig. 1). The term “organoid,” meaning “resembling an organ,” refers to culture systems in which cells self-organize into 3D tissues that recapitulate—to at least some degree—the cellular diversity, 3D spatial organization, and functional properties of native organs. Researchers have developed protocols for generating intestinal (Spence et al. 2011), cerebral (Lancaster et al. 2013), retinal (Eiraku et al. 2011; Nakano et al. 2012), kidney (Freedman et al. 2015; Morizane et al. 2015; Takasato et al. 2015), lung (Dye et al. 2015), and—of course—inner ear (Koehler et al. 2013, 2017) organoids from PSCs. LGR5^+^ tissue-specific progenitor cells have also been expanded into 3D in vitro tissues resembling pyloric epithelium (Barker et al. 2010), small intestinal crypt-villus units (Sato et al. 2009), colonic crypts (Sato et al. 2011), and hepatocytes (Huch et al. 2013). A population of LGR5^+^ support cells that displays some characteristics of tissue-specific progenitors is also present in the mammalian cochlea (Shi et al. 2012). McLean et al. (2017) recently described a protocol for expanding these LGR5^+^ support cells in vitro into 3D vesicles lined by hair cell- and support cell-like cells. However, these LGR5^+^ support cell-derived vesicles fail to recapitulate the full cellular diversity of the mammalian cochlea.

PSC-derived inner ear organoids, in contrast, comprise not only hair cell- and support cell-like cells, but also neuron-like cells and distinct regions of PAX8^+^ non-sensory otic-like epithelium and TFAP2A^+^/SLUG^+^ periotic-like mesenchyme (Bouchard et al. 2010; Koehler et al. 2013, 2017). To date, all publications describing the successful generation of PSC-derived inner ear organoids have built upon the foundations of the stepwise induction protocol described by Koehler et al. (2013) (see DeJonge et al. 2016; Hartman et al. 2018; Koehler et al. 2017; Liu et al. 2016; Perny et al. 2017; Schaefer et al. 2018; Tang et al. 2019). PSC-derived aggregates are treated with BMP-4 and the TGF-beta inhibitor SB-431542, which promote the specification of non-neural ectoderm, while simultaneously inhibiting the formation of mesendoderm. Next, FGF-2 and LDN-193189, a BMP-4 inhibitor, are used to drive non-neural ectoderm toward a pre-placodal, rather than an epidermal fate. Under the influence of endogenous Wnt signaling (DeJonge et al. 2016; Koehler et al. 2013), the pre-placodal ectoderm-like tissues adopt an otic placodal fate, and mimicking the sequential genesis of the otic pit and vesicle, invaginate to form distinct vesicles (Koehler et al. 2013, 2017). DeJonge et al. (2016) later discovered that otic placodal differentiation could be enhanced by exogenous Wnt signaling through addition of the GSK3 inhibitor CHIR-99021. After a period of self-directed maturation, the vesicles contain luminal patches of MYO7A^+^ hair cell-like cells, which abut a dense, basal layer of SOX2^+^ support cell-like cells and form putative synapses with TUJ1^+^ neuron-like cells (Koehler et al. 2013, 2017).

The further, intensive characterization of inner ear organoids by Koehler et al. (2013, 2017) and others has revealed an impressive similarity to native inner ear tissues. Besides *Myo7a*, PSC-derived hair cell-like cells were found to express a number of other hair cell-specific genes, including *Pou4f3* (Koehler et al. 2013, 2017; Xiang et al. 1998, 2003), *Otof* (Tang et al. 2019; Yasunaga et al. 1999), and *Tmc1* (Kurima et al. 2002; Tang et al. 2019). The majority of the hair cell-like cells also labeled for a handful of proteins expressed in the type II vestibular hair cells—but not in the inner, outer, or type I hair cells—of adult mice, namely CALB2 (Desai et al. 2005; Koehler et al. 2013, 2017), SOX2 (Koehler et al. 2013, 2017; Oesterle et al. 2008), and ANXA4 (Koehler et al. 2017; Liu et al. 2016; McInturff et al. 2018). Consistent with this, most hair cells in mouse inner ear organoids also adopted the morphology of type II vestibular cells, as well as voltage response, fast inward rectifier, and large outward delayed rectifier currents that resembled those of the type II vestibular hair cells of the postnatal day 4 (P4) mouse utricle (Liu et al. 2016). Amazingly, these currents underwent a maturation process similar to that in the native mouse utricle. That is, the prevalence of hyperpolarization-activated cation channels increased after culture day 22, while that of voltage-dependent Na^+^ currents declined (Liu et al. 2016).

In addition, the hair cell-like cells in inner ear organoids seem to have the ‘necessary parts’ for mechanotransduction. PSC-derived hair cell-like cells display F-actin^+^/ESPN^+^ stereocilia bundles, PCDH15^+^/CDH23^+^ tip link-like structures, and a single acetylated-alpha-tubulin^+^ kinocilium on their luminal surfaces, reminiscent of the mechanotransduction apparatus of native vestibular hair cells (Koehler et al. 2013; Tang et al. 2019). The hair bundle-like structures of these cells often demonstrated a pattern of local alignment that was reminiscent of the organization seen in the mouse utricle (Liu et al. 2016), and by day 24, the length of stereocilia on mouse PSC-derived hair cells fell within the normal range for adult mouse utricular hair cells. FM4-64 and FM1-43 uptake assays suggested the presence of functional mechanosensitive channels (Koehler et al. 2013; Liu et al. 2016), which was confirmed by the measurement of mechanotransduction currents by day 25 (Liu et al. 2016). Furthermore, CTBP2^+^ punctae were observed at the base of hair cell-like cells in close proximity to the neurite-like extensions of neuron-like cells, which, in turn, expressed multiple postsynaptic markers (Koehler et al. 2013, 2017). Hair cell-like cells also exhibited the depolarization-activated Ca2^+^ currents necessary for neurotransmitter release in vivo (Liu et al. 2016).

## Modeling hereditary deafness in 2D and 3D culture

**Fig. 2:**
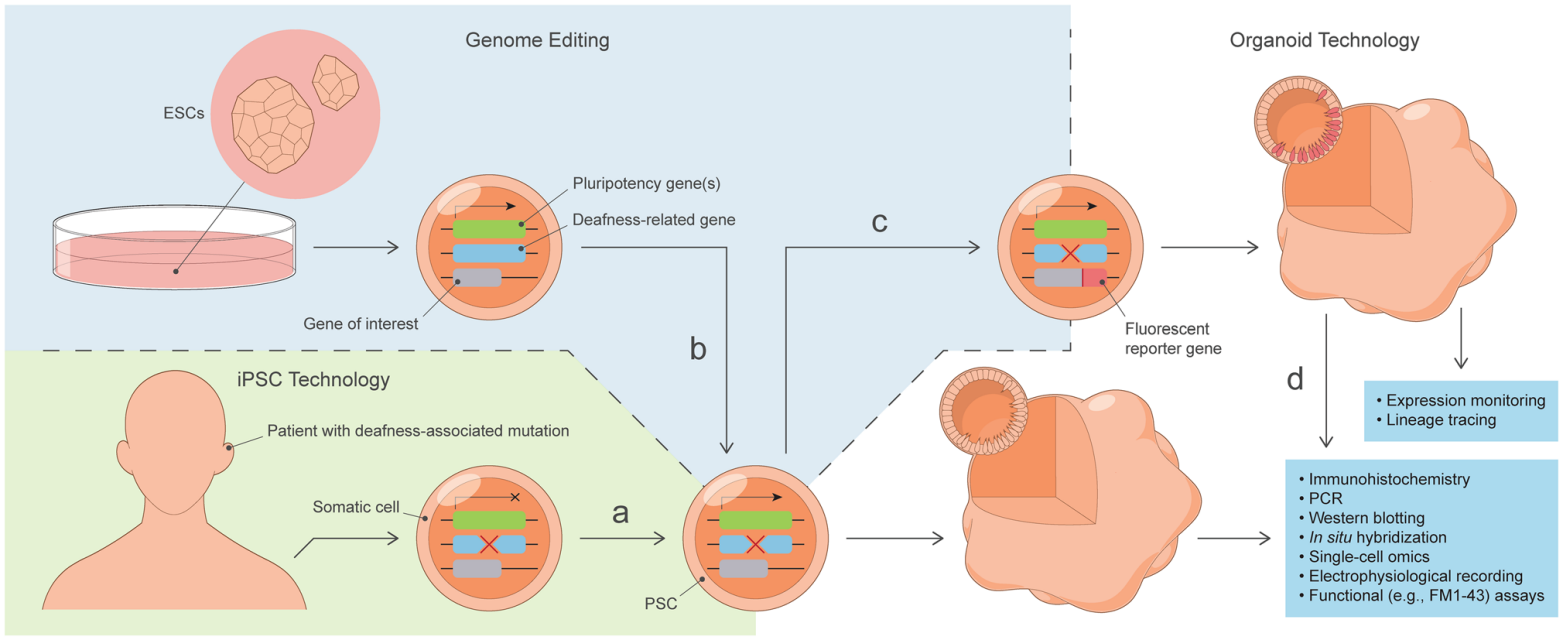
Graphical representation of the generation and analysis of PSC-derived inner ear organoid models of hereditary deafness. **a** Patient-specific iPSCs with mutation in deafness-related gene (X) are generated from somatic cells through induced expression of pluripotency genes. **b** Precision genome editing is used to create targeted, deafness-related mutations in ESCs. **c** The coding sequence of a fluorophore (e.g., tdTomato) is inserted downstream of the promoter for a gene-of-interest (e.g., deafness-related gene, cell type-specific gene, or regionally expressed transcription factor) to generate a fluorescent reporter PSC line. **d** Fluorescent cells within organoids can be selectively harvested, either manually or automatically (e.g., by FACS), for further analyses. *ESCs* embryonic stem cells, *FACS* fluorescence-activated cell sorting, *iPSC* induced pluripotent stem cell

An estimated 80% of prelingual deafness in the developed world is thought to be attributable to genetic causes (Shearer et al. 1993). Modeling hereditary deafness, therefore, represents an incredibly valuable application of PSC-derived inner ear organoids. There are two general approaches to modeling hereditary deafness in vitro (Fig. 2). The first approach involves the targeted introduction of deafness-associated mutations into wild-type ESC lines via CRISPR-Cas9 (Cong et al. 2013; Mali et al. 2013), prime editing (Anzalone et al. 2019), or another precision genome-editing technique. The second is to harvest somatic cells—often dermal fibroblasts or peripheral blood mononuclear cells—from patients with hereditary deafness and convert them into induced PSCs (iPSCs) (Takahashi et al. 2007; Takahashi and Yamanaka 2006). Stepwise induction protocols can then be used for the directed differentiation of inner ear-like tissues from iPSCs or CRISPR-Cas9-edited ESCs or iPSCs.

iPSCs certainly hold more therapeutic potential than ESCs, as iPSC-derived donor cells can be used for autologous cell-based inner ear therapy without concern for rejection. However, several studies have revealed the presence of significant genetic background variation among iPSCs, which, in turn, leads to significant variability in the directed differentiation process (Burrows et al. 2016; Kyttala et al. 2016; Rouhani et al. 2014). Indeed, DeJonge et al. (2016) reported that the optimal timing of FGF-2/LDN-193189 treatment in the stepwise induction of inner ear organoids varied among four miPSC lines, while Koehler et al. (2017) found that exogenous BMP-4 was necessary for the induction of non-neural ectoderm in hiPSC-, but not hESC-, derived aggregates. In contrast, in the absence of off-target mutations, genetically engineered ESCs have a homogenous genetic background, allowing for the more consistent derivation of inner ear organoids, as well as the ability to control for genetic background noise when comparing wild-type and mutant PSC-derived cells or tissues. Another limitation of iPSCs is the rarity of specific single-gene mutations, which are often only reported in individual families. The routine use of iPSC-based culture systems in modeling hereditary deafness will, therefore, likely require a significant collaborative effort with the establishment of an iPSC biorepository.

Nonetheless, researchers have used hiPSC-based, 2D culture systems to model two forms of autosomal recessive non-syndromic deafness, DFNB2 (Tang et al. 2016) and DFNB3 (Chen et al. 2016a), as well as Pendred syndrome (Hosoya et al. 2017, 2019), the inherited peripheral neuropathy Charcot–Marie–Tooth disease type 1A (CMT1A) (Shi et al. 2018), and myoclonic epilepsy with ragged-red fibers (MERRF) (Chen et al. 2018), a mitochondrial disorder characterized by SNHL, myopathy, ataxia, and—as the name implies—epilepsy. Shi et al. (2018) generated iPSCs from CMT1A and healthy patients, and then differentiated these cells into neural crest stem cell-like cells in 2D culture. Under the appropriate conditions, CMT1A and wild-type neural crest stem cell-like cells were able to differentiate into osteoblast-, adipocyte-, chondrocyte-, smooth muscle cell-, and neuron-like cells with similar efficiency. However, under conditions in which wild-type cells gave rise to GFAP^+^/S100B^+^ Schwann cell-like cells, CMT1A hiPSC-derived neural crest stem cell-like cells instead produced CD34^+^ endoneurial fibroblast-like cells, suggesting that the pathogenesis of CMT1A may be related to the aberrant differentiation of Schwann cell progenitors. Hosoya et al. (2017) went a step further, not only modeling Pendred syndrome in an iPSC-based 2D culture, but also using this model to test the therapeutic effects of low-dose rapamycin and metformin. This iPSC-based Pendred syndrome model has since been used to determine the minimum effective dose of rapamycin in preventing otic-like cell death in vitro (Hosoya et al. 2019).

Several other studies have used CRISPR-Cas9 genome editing to generate in vitro models of genetic deafness. *Barhl1* is a mammalian homologue of the *Drosophila* homeobox gene *BarH1* (Bulfone et al. 2000). *Barhl1* encodes a homeodomain transcription factor expressed in the inner ear and central nervous system. While there is no human deafness phenotype currently associated with *BARHL1* mutation, *Barhl1*-null mice exhibit progressive SNHL (Li et al. 2002). Zhejiang University scientists have used CRISPR-Cas9 technology to generate frameshift mutations in the coding region (Zhong et al. 2018) and 3’ enhancer (Hou et al. 2019) of the *Barhl1* gene in mESCs. To investigate the underlying mechanism of SNHL in *Barhl1* mutants, both wild-type and *Barhl1*-mutant mESCs were subjected to a stepwise induction protocol for deriving hair cell-like cells in 2D culture. The authors used several analytical methods to investigate the effects of *Barhl1* mutation on hair cell differentiation, revealing a significant downregulation of hair cell-specific genes in both mutant cell lines (Hou et al. 2019; Zhong et al. 2018). Downregulated hair cell-specific genes were then cross-referenced with potential BARHL1 targets (Zhong et al. 2018). The results of this analysis suggested that the effects of *Barhl1* mutation on hair cell differentiation may be mediated by the downregulation of *Clic5* and *Ush1g*.

Similarly, Tang et al. (2019) used CRISPR-Cas9 to generate *Tmprss3*-KO mESCs. *Tmprss3* encodes a type II transmembrane serine protease. Mutations in *TMPRSS3* are the cause of autosomal recessive non-syndromic deafness DFNB8/10, which is characterized by prelingual SNHL (Scott et al. 2001). Mice homozygous for the nonsense mutation *Tmprss3*^*Y260X*^ exhibit rapid hair cell degeneration at P12 following a period of normal hair cell development (Fasquelle et al. 2011). Despite the presence of this mouse model, the exact functional role of TMPRSS3 in the inner ear remains poorly understood, and the precise pathophysiologic mechanism underlying DFNB8/10 has yet to be revealed. Thus, *Tmprss3-*KO and wild-type mESCs were subjected to directed differentiation into inner ear organoids (Tang et al. 2019). The researchers found that, while *Tmprss3*-KO PSCs initially gave rise to hair cell-like cells with normal hair bundles and FM1-43 uptake, by day 38 (equivalent to P12-P14 in vivo), *Tmprss3-*KO hair cell-like cells exhibited significantly higher levels of the apoptosis protein caspase-3. The organoid format permitted easy access to inner ear-like tissues for single-cell RNA sequencing (scRNA-seq), which revealed potential roles for calcium ion homeostasis and extracellular matrix maintenance in TMPRSS3-related deafness.

## Unsettled questions in inner ear organoid research

Despite the promise of inner ear organoids in modeling human deafness, we must exercise caution when applying findings in inner ear organoids to the human auditory system. A recent scRNA-seq analysis revealed that the specification of distinct cellular subtypes was not achieved in cortical organoid culture and suggested that this was due, at least in part, to high levels of endoplasmic reticulum (ER) stress (Bhaduri et al. 2020). Only time will tell if this ER stress-induced inhibition of cell type specification is an inherent feature of organoid culture or simply a matter of optimizing culture conditions, or—for that matter—whether these results are even generalizable to organoids on the whole. It is certainly auspicious for the future of inner ear organoid research that many deafness-related genes are expressed in inner ear organoids with a similar spatiotemporal pattern to the native inner ear (Table 1), while many others have been detected in putative otic-like sensory epithelial cells by scRNA-seq (Tang et al. 2019). Nonetheless, the process of characterizing inner ear organoids is still in its infancy, and establishing the inner ear organoid as a valid model of the human auditory system in health, disease, and development will require the continued dedication and collaboration of stem cell biologists, cellular electrophysiologists, bioinformaticians, and others. We will use this opportunity to suggest and discuss areas for the further, intensive characterization of inner ear organoids.

**Table 1:** Summary of the available spatiotemporal expression data for deafness-related genes (column 1) in inner ear organoids (column 2) and the rodent inner ear (column 3)

Gene	Expression pattern in inner ear organoids	In vivo expression pattern	Disorder	Clinical findings
*ACTG1*	Expressed in mouse otic-like epithelium and hair bundle-like structures by day 20 (Koehler et al. 2013; Liu et al 2016; Perny et al. 2017; Schaefer et al. 2018; Tang et al. 2019); expressed in human otic-like epithelium and hair bundle-like structures by day 60 (Koehler et al. 2017)	Expressed in the stereocilia, cuticular plates, and adherens junctions of mouse HCs by E16.5 (Andrade 2015)	DFNA20/26	Progressive postlingual high-frequency SNHL (van Wijk et al. 2003; Zhu et al. 2003)
*CDH23*	Expressed in mouse hair bundle-like structures by day 23 (Tang et al. 2019)	Expressed in mouse cochlear and vestibular HCs as early as E16.5^a^ and E14.5, respectively, persists to at least P16; expressed in Reissner’s membrane from E16.5 onward (Lagziel et al. 2005)	Usher syndrome type 1D	Profound congenital SNHL, vestibular dysfunction, retinitis pigmentosa (Bolz et al. 2001; Bork et al. 2001)
			DFNB12	Profound congenital SNHL (Bork et al. 2001)
*ESPN*	Expressed in mouse hair bundle-like structures by day 20 (DeJonge et al. 2016; Hartman et al. 2018; Koehler et al. 2013; Liu et al. 2016; Tang et al. 2019); expressed in human hair bundle-like structures by day 70 (Koehler et al. 2017)	Expressed in the rat otic pit, vesicle, and epithelium by E10, enriched in vestibular, inner, and outer HCs as early as E14, E16, and E18, respectively^a^ (Sekerkova et al. 2006)	DFNB36	Profound prelingual SNHL, vestibular dysfunction (Naz et al. 2004)
*EYA1*	Expressed in mouse otic-like vesicles at day 12 (Schaefer et al. 2018)	Expressed in the ventral aspect of the mouse otic vesicle at E9.5–E11.5; expressed in the otic sensory epithelium from E12.5 to birth, restricted to vestibular HCs and cochlear sensory epithelium by P20; expressed in the vestibulocochlear ganglion by E10.5, persists to at least P20; expressed in the vestibular transitional epithelium from E15.5 to E17.5, the saccular roof from E15.5 to birth, and the spiral ligament at P8 (Kalatzis et al. 1998; Xu et al. 2021; Zheng et al. 2003)	Branchio-oto-renal spectrum disorders	Sensorineural, conductive, or mixed HL, branchial arch anomalies, renal/urinary tract abnormalities (Abdelhak et al. 1997; Vincent et al. 1997)
*MYO6*	Expressed in mouse HC-like cells by day 17 (Hartman et al. 2018)	Expressed in mouse HCs as early as E13.5 (Xiang et al. 1998)	DFNA22	Progressive postlingual high-frequency SNHL (Melchionda et al. 2001)
			DFNB37	Severe-to-profound congenital SNHL, vestibular dysfunction (Ahmed et al. 2003a)
*MYO7A*	Expressed in mouse otic-like vesicles at day 14, restricted to HC-like cells by day 15 (DeJonge et al. 2016; Hartman et al. 2018; Koehler et al. 2013; Liu et al. 2016; Perny et al. 2017; Schaefer et al. 2018; Tang et al. 2019); expressed in human HC-like cells by day 60 (Koehler et al. 2017)	Expressed in mouse HCs as early as E13.5 (Xiang et al. 1998)	Usher syndrome type 1B	Profound congenital SNHL, vestibular dysfunction, retinitis pigmentosa (Weil et al. 1995)
			DFNA11	Progressive postlingual SNHL (Liu et al. 1997b)
			DFNB2	Profound SNHL, vestibular dysfunction (Liu et al. 1997a; Weil et al. 1997)
*PCDH15*	Expressed in mouse hair bundle-like structures by day 23 (Tang et al. 2019)	Expressed in the mouse cochlear HCs by P3^a^; expressed in vestibular HCs as early as E15.5 (Ahmed et al. 2006)^b^	Usher syndrome type 1F	Profound congenital SNHL, vestibular dysfunction, retinitis pigmentosa (Ahmed et al. 2001; Alagramam et al. 2001)
			DFNB23	Severe-to-profound prelingual SNHL (Ahmed et al. 2003b)
*POU4F3*	Expressed in mouse HC-like cells by day 16 (DeJonge et al. 2016; Koehler et al. 2013; Tang et al. 2019); expressed in human HC-like cells by day 140 (Koehler et al. 2017)	Expressed in mouse HCs as early as E12.5 (Xiang et al. 1998)	DFNA15	Progressive postlingual high-frequency SNHL, vestibular dysfunction (Vahava et al. 1998)
*SIX1*	Expressed in the outer epithelium of mPSC-derived aggregates at d6, restricted to otic-like vesicles at day 12 (Koehler et al. 2013; Schaefer et al. 2018)	Expressed in the ventral aspect of the mouse otic pit and vesicle from E8.75 to E10.5, expressed in the vestibular sensory epithelium and greater and lesser epithelial ridges at E12.5, restricted to the vestibular and cochlear HCs,^a^ greater epithelial ridge, and stria vascularis as early as E15.5; expressed in the vestibulocochlear ganglion at E12.5 (Xu et al. 2021; Zheng et al. 2003)	Branchio-oto-renal spectrum disorders	Sensorineural, conductive, or mixed HL, branchial arch anomalies, renal/urinary tract abnormalities (Ruf et al. 2004)
			DFNA23	Sensorineural or mixed HL (Mosrati et al. 2011)
*SOX10*	Expressed in human otic-like pits and vesicles at days 14–35, restricted to SC-like cells and non-sensory otic-like epithelium by day 75; expressed in cranial neural crest-like cells at day 35 (Koehler et al. 2017)	Expressed in the mouse otic pit, vesicle, and epithelium from E9.0 to E16.5, restricted to the SCs and non-sensory otic epithelium by P1, persists to at least P21, except in tympanic border cells; expressed in migrating cranial neural crest cells from E10.5 to E14.5, persists in cranial neural crest derivatives (intermediate and glial cells) to at least P21 (Wakaoka et al. 2013)	Waardenburg syndrome type 2E	Profound prelingual SNHL, pigmentation abnormalities (Bondurand et al. 2007)
			Waardenburg syndrome type 4C	Profound congenital SNHL, pigmentation abnormalities, Hirschsprung disease (Pingault et al. 1998)

The associated genetic disease(s) (column 4) and its/their clinical findings (column 5) are provided for the reader’s reference

*HC* hair cell, *HL* hearing loss, *mPSC* mouse pluripotent stem cell, *P* postnatal day, *SC* support cell, *SNHL* sensorineural hearing loss

^a^HC-specific gene expression commences in a basal-to-apical direction along the cochlear duct, with the embryonic date (E) provided here denoting onset at the cochlear base

^b^PCDH15 has several isoforms with distinct spatiotemporal expression patterns

Perhaps the single greatest open question in inner ear organoid research—which is also a significant limitation of inner ear organoids—relates to the conspicuous absence of hair cell-like cells with *a cochlear phenotype*. Indeed, until cochlear-type inner ear organoids are derived, these 3D culture systems will never be able to fully recapitulate the human auditory system. There are several possible explanations for the notable absence of cochlear hair cell-like cells in organoid culture. First, the non-physiologic culture environment may be causing ER stress that, in turn, inhibits cell type specification, as observed in cortical organoids (Bhaduri et al. 2020). It is not exactly clear why ER stress would specifically inhibit the differentiation of cochlear hair cell-like cells. However, it is worth noting that vestibular hair cells express many hair cell-specific genes at earlier developmental timepoints than cochlear hair cells (Table 1) and share several features with immature hair cells that are eventually lost in cochlear hair cells, including the presence of a kinocilium and—in type II hair cells—expression of SOX2. This suggests that the differentiation of vestibular hair cells may require less molecular specification of hair cell progenitors than cochlear hair cells. Another possibility is that the innervation by PSC-derived neuron-like cells in inner ear organoids is too limited to provide adequate trophic support for the survival of early cochlear hair cell-like cells. Indeed, Kersigo and Fritzsch (2015) elegantly demonstrated that denervation results in the progressive loss of hair cells in mice, but that vestibular hair cells are significantly more resilient. It is, therefore, possible that nascent cochlear hair cell-like cells are originally present in inner ear organoids but that inadequate innervation by neuron-like cells eventually leads to their preferential loss over vestibular hair cell-like cells.

Alternatively, it may be that current protocols produce a signaling environment favoring the differentiation of vestibular hair cell-like cells from hair cell progenitor-like cells. A series of elegant studies has demonstrated that development of the cochlea and vestibule are regulated by the opposing effects of ventral and dorsal signals. Sonic hedgehog (SHH) induces expression of ventral transcription factors such as *Otx2*, *Pax2*, and *Ngn1*, while inhibiting expression of the dorsal marker *Dlx5* (Riccomagno et al. 2002). Conversely, Wnt and BMP both induce the expression of *Dlx5*, and BMP inhibits *Otx2* (Ohta et al. 2016; Riccomagno et al. 2005). These signals are so critical to the specification of the cochlea and vestibule that their loss or ectopic expression has been shown to produce dramatic malformations. For example, the loss of SHH results in complete absence of the cochlear duct in mice (Riccomagno et al. 2002). Thus, the preferential differentiation of vestibular hair cell-like cells could be explained by the relative overactivity of dorsal signals, and therefore, corrected by the precisely timed addition of a ventralizing molecule, such as the SHH agonist purmorphamine, or an inhibitor of dorsal signals, such as LDN-193189. Jeong et al. (2018) recently reported the generation of inner ear organoids with hair cell-like cells that expressed some markers of cochlear hair cells. Notably, this was achieved with only slight modifications to the protocol described by Koehler et al. (2013), such as the maintenance of PSCs on feeder cells. However, the purported cochlear hair cell-like cells were incompletely characterized, and the result has yet to be replicated. If these results are replicated, it will be important to investigate the mechanism(s) by which the modified protocol produced cochlear-type hair cell-like cells.

*Cell–cell junctions*, including gap junctions, tight junctions, and tricellular junctions, are broadly present in the membranous labyrinth of the mammalian inner ear (Forge et al. 1999; Kitajiri et al. 2004) and are essential in maintaining the functional barrier between the perilymphatic and endolymphatic compartments, as well as facilitating neuronal growth and promoting cellular organization (Reviewed in Jagger and Forge 2015; Kitajiri and Katsuno 2016). Indeed, the cell–cell junctions of the inner ear are essential for normal hearing, evidenced by the fact that deficiencies in several junctional proteins are associated with hereditary deafness (Shearer et al. 1993; Toriello and Smith 2013), including GJB2 (connexin 26) deficiency (DFNB1A), the most common cause of autosomal recessive non-syndromic deafness in humans. Phalloidin staining has revealed the presence of an apical F-actin network in inner ear organoids, reminiscent of the network of cell–cell tight junctions between support cells of the mouse inner ear (Koehler et al. 2013; Schaefer et al. 2018), and scRNA-seq data have revealed the expression of *Cldn9*, which encodes the tight junction protein claudin-9, in putative mouse hair cell-like cells (Tang et al. 2019). However, the subcellular co-localization of F-actin, claudins, and other tight junction proteins, such as occludins and junctional adhesion molecules, at cell–cell interfaces has yet to be demonstrated in inner ear organoids. The extent to which inner ear organoids recapitulate the many, highly specialized, *non-neurosensory cell types* of the native inner ear is another open question in inner ear organoid research. However, this subject was recently discussed in great depth by van der Valk et al. (2021), and we will direct the interested reader to this excellent review.

Should inner ear organoids prove to accurately recapitulate the structural, functional, and molecular features of the native inner ear, then it is reasonable to assume that the same *extrinsic insults* that lead to SNHL in humans would be similarly deleterious to inner ear organoids, allowing researchers to study the pathophysiologic mechanisms by which they act. However, it is not obvious how certain causes of SNHL (e.g., noise-induced hearing loss) could be simulated in the culture environment, while limits on culture duration will likely preclude any meaningful study of non-genetic, age-related hearing loss. Nonetheless, a wide range of chemical and infectious insults remain potentially amenable for study in inner ear organoids. To date, few studies have focused on the ability of inner ear organoids to model these insults. The ototoxic aminoglycoside dihydrostreptomycin has been shown to reversibly block stimulus-evoked currents in the hair cell-like cells of mouse organoids (Liu et al. 2016), while another aminoglycoside, gentamicin, failed to produce any obvious loss of hair bundle-like structures (Schaefer et al. 2018). Further studies are needed to better characterize inner ear organoids’ susceptibility to aminoglycoside antibiotics and other extrinsic insults known to cause SNHL.

## Genome editing and single-cell omics in inner ear organoids

Genome editing encompasses a number of technologies that allow researchers to alter the genetic code of a cell or organism. Genome-editing technologies have become increasingly targeted over the years. For example, fluorescent reporter PSC lines are generated by the targeted insertion of a fluorescent protein coding sequence downstream of the promoter of a gene-of-interest, which—in the case of inner ear organoids—could be a deafness-related gene, cell type-specific gene, or regionally expressed transcription factor. PSC reporter lines for PAX2, FBXO2, and ATOH1, have all been generated and differentiated into inner ear organoids (DeJonge et al. 2016; Hartman et al. 2018; Koehler et al. 2017; Liu et al. 2016; Schaefer et al. 2018), with numerous applications. For example, Liu et al. (2016) used the eGFP signal emitted by the hair cell-like cells of *Atoh1*-eGFP PSC-derived aggregates to selectively harvest hair cell-like cell bearing vesicles for electrophysiological recording. FACS sorting of organoid cells derived from reporter lines could be similarly used for the selective isolation of otic-like cell types for single-cell analyses. Fluorescent reporters can also been used for expression monitoring, allowing researchers to observe the expression patterns of developmental transcription factors or cell type-specific marker genes over the course of organoid development. For example, Hartman et al. (2018) used a Venus reporter for *Fbxo2*, an otic lineage-specific gene, to demonstrate surprising differences in spatiotemporal expression patterns between the native mouse vestibular epithelium and mouse inner ear organoids. Another application of fluorescent reporters is lineage tracing. Chimeric inner ear organoids could be generated from a mixture of mutant and wild-type cells to study the role of paracrine signaling in different types of hereditary deafness or to control for batch-to-batch variability in comparisons of wild-type and mutant cells. Combining this technology with fluorescent reporter lines would allow for the easy identification of genotype in chimeric organoids via fluorescent microscopy or single-cell transcriptomics.

scRNA-seq has a number of applications in both auditory research (Reviewed in Pyle and Hoa 2020) and PSC-derived organoids (Reviewed in Camp and Treutlein 2017; Qin and Tape 2020), and will undoubtedly facilitate the further characterization of organoids and their application in disease modeling. Several unsettled questions in inner ear organoid research could be answered by the generation of a single-cell transcriptional atlas (Kolla et al. 2020; Korrapati et al. 2019; Petitpre et al. 2018; Shrestha et al. 2018; Sun et al. 2018) for inner ear organoids or by scRNA-seq analyses that focus on the expression of signaling molecules, stress response genes, or regionally expressed transcription factors. scRNA-seq has already been used to compare the transcriptional profiles of mutant and wild-type inner ear organoids in the hopes of elucidating the underlying molecular mechanisms of hereditary deafness Tang et al. (2019). Meanwhile, single-cell RT-qPCR, has been used to compare the gene expression between PSC-derived otic-like tissues and the native inner ear to investigate the transcriptional fidelity of a 2D culture system and optimize the protocol (Ealy et al. 2016). In the future, comparative analyses could be performed to investigate transcriptional heterogeneity among organoids derived from different cell lines or according to different protocols, as has been demonstrated in kidney organoids (Wu et al. 2018). Other single-cell technologies, such as scATAC-seq (single-cell assay for transposase-accessible chromatin with high-throughput sequencing), could be similarly applied to compare, for example, the epigenetic landscape in inner ear organoids and the native inner ear. Multiple single-cell technologies can also be simultaneously applied (single-cell multi-omics), and the resulting data integrated for analysis with computational tools such as Seurat v3 (Stuart et al. 2019) or LIGER (Welch et al. 2019). Protocols have even been developed which would allow for simultaneous characterization of the transcriptome, electrophysiology, and/or morphology of single PSC-derived hair cell- and neuron-like cells (Bardy et al. 2016; Cadwell et al. 2016; Chen et al. 2016b; Foldy et al. 2016; Fuzik et al. 2016; Ranum et al. 2019), permitting the remarkably detailed comparison of native and PSC-derived cells, and possibly, the identification of cellular subtypes in inner ear organoids.

## Translational applications of inner ear organoids

The potential translational applications of inner ear organoids in disease modeling are incredibly numerous. Should inner ear organoids prove to be sensitive to the ototoxic compounds, they would be incredibly useful in the scalable, high-throughput testing of drug toxicity in pre-clinical trials. Conversely, when combined with developmental or regeneration studies, this same scalability could be applied toward the large-scale, high-throughput, pre-clinical screening of otoprotective or oto-regenerative compounds. Inner ear organoids could also serve as a platform for testing the effects of CRISPR-Cas9 genetic correction of single-gene mutations. This could even one day be employed in a patient-specific manner, with the generation of inner ear organoids from CRISPR-Cas9-corrected patient-specific iPSCs being a routine quality check before cell-based therapy is performed for SNHL. Lastly, inner ear organoid models of inner ear disease could be used for the testing of emerging therapies such as cell-based and gene therapy. Successfully employing inner ear organoids for these applications will almost certainly require that researchers find a way to overcome the previously reported high variability and low efficiency of organoid generation (Schaefer et al. 2018; Koehler et al. 2017), as well as the overgrowth of periotic-like mesenchyme in late-stage culture, which hinders analytic methods such as expression monitoring, whole-mount imaging, and single-cell isolation. Fortunately, progress toward these goals is already underway. For example, Chang et al. (2020) recently reported increased efficiency of inner ear organoid generation with the use of photobiomodulation and hanging droplet techniques, while Hocevar et al. (2021) described a method for dissecting organoids away from their aggregates and reported that, when cultured in media with Matrigel, organoids display the same autonomy seen in vivo (Swanson et al. 1990).

## Summary

Since the human inner ear cannot be biopsied without causing significant, irreversible damage to the hearing or balance organ, the biological study of human inner ear cells has traditionally been limited to scarce fetal and cadaveric tissues. Researchers recently described a protocol for generating inner ear organoids, which could serve as a scalable, high-fidelity alternative to animal models. However, many questions and challenges remain, including how cochlear-type hair cell-like cells can be derived, whether cell–cell junctions are present, whether non-neurosensory inner cell types are represented, and whether inner ear organoids are susceptible to the same extrinsic insults that cause deafness in humans and other mammals. With the continued dedication of stem cell biologists, cellular electrophysiologists, and bioinformaticians, and the utilization of fluorescent reporter lines and single-cell omics, it is likely that these questions and others will be answered in the coming years. If, through these efforts, high-fidelity, human cochlear inner ear organoids are successfully generated, then a wide array of translational applications await inner ear organoids, including high-throughput drug and toxicity screens and pre-clinical testing and patient-specific quality checks for stem cell and gene therapies.

## Data Availability

Not applicable.
